# Biological activity of human IgE monoclonal antibodies targeting Der p 2, Fel d 1, Ara h 2 in basophil mediator release assays

**DOI:** 10.3389/fimmu.2023.1155613

**Published:** 2023-05-09

**Authors:** Glorismer Pena-Castellanos, Bryan R. E. Smith, Anna Pomés, Scott A. Smith, Maria A. Stigler, Hannah L. Widauer, Serge A. Versteeg, Ronald van Ree, Martin D. Chapman, Lorenz Aglas

**Affiliations:** ^1^ Department of Biosciences and Medical Biology, University of Salzburg, Salzburg, Austria; ^2^ InBio, Charlottesville, VA, United States; ^3^ Department of Medicine, Vanderbilt University Medical Center, Nashville, TN, United States; ^4^ Department of Experimental Immunology, Amsterdam University Medical Centers, Amsterdam, Netherlands; ^5^ Department of Otorhinolaryngology, Amsterdam University Medical Centers, Amsterdam, Netherlands

**Keywords:** human IgE monoclonal antibodies, mediator release assay, Der p 2, Fel d 1, Ara h 2, allergy diagnosis

## Abstract

**Background:**

Human Immunoglobulin E monoclonal antibodies (hIgE mAb) are unique tools for investigating IgE responses. Here, the biological activity of hIgE mAb, derived from immortalized B cells harvested from the blood of allergic donors, targeting three allergens (Der p 2, Fel d 1 and Ara h 2) was investigated.

**Methods:**

Three Der p 2-, three Fel d 1- and five Ara h 2-specific hIgE mAb produced by human B cell hybridomas, were combined in pairs and used to passively sensitize humanized rat basophilic leukemia cells and compared with sensitization using serum pools. Sensitized cells were stimulated with corresponding allergens (recombinant or purified), allergen extracts or structural homologs, having 40-88% sequence similarity, and compared for mediator (β-hexosaminidase) release.

**Results:**

One, two and eight pairs of Der p 2-, Fel d 1- and Ara h 2-specific hIgE mAb, respectively, produced significant mediator release (>50%). A minimum hIgE mAb concentration of 15-30 kU/L and a minimum antigen concentration between 0.01-0.1 µg/mL were sufficient to induce a pronounced mediator release. Individual sensitization with one Ara h 2-specific hIgE mAb was able to induce crosslinking independently of a second specific hIgE mAb. Der p 2- and Ara h 2-specific mAb showed a high allergen specificity when compared to homologs. Mediator release from cells sensitized with hIgE mAb was comparable to serum sensitization.

**Conclusion:**

The biological activity of hIgE mAb reported here provides the foundation for novel methods of standardization and quality control of allergen products and for mechanistic studies of IgE-mediated allergic diseases, using hIgE mAb.

## 1 Introduction

In one of the most recent developments of hybridoma technology, unique human IgE monoclonal antibodies (hIgE mAb) have been derived for the first time from B cells from allergic patients (1, 2). Producing hIgE mAb was previously not possible because of the low amounts of IgE antibodies in serum (<1 µg/mL) and the low frequency of IgE-secreting B cells in the peripheral blood. The new approach involves immortalizing enriched IgE-secreting B cells *via* electrical cytofusion with a non-secreting myeloma cell line. IgE-secreting human hybridomas are subsequently screened for specificity, selected, cloned and expanded. Secreted hIgE mAb, expressed in serum-free medium, are purified *via* anti-IgE immunoaffinity chromatography (3). Using this approach, panels of hIgE mAb with naturally occurring pairing of IgE heavy and light chains have been developed (which overcame a limitation of engineered recombinant antibodies). Exploiting this hybridoma technology, hIgE mAb specific for the house dust mite (HDM) *Dermatophagoides pteronyssinus* allergen Der p 2 were produced for IgE epitope mapping (4). These Der p 2-specific hIgE mAb, were tested in pairs to induce anaphylaxis in mice (4). From the production of these Der p 2-specific hIgE mAb came the first ever high-resolution structure of a conformational human IgE epitope on Der p 2 (5).

In addition to structural investigations, hIgE mAb have also been used to determine the quantity of specific allergens in extracts using *Aspergillus*-specific hIgE mAb (1). Previous studies investigating the biological activity on IgE antibodies based on basophil degranulation were performed using either human polyclonal IgE purified from patient´s serum (6, 7), recombinant chimeric IgE (8), or murine IgE mAb (9). Within the past three years, panels of hIgE mAb to indoor allergens and to food allergens have been produced. The hIgE mAb can be produced at a high concentration (>50,000 kU/L) and are high-affinity antibodies directed against multiple epitopes with strong potential to advance the field of allergy/immunology.

We present herein for the first time a complete characterization of fully human IgE_mAb, derived from immortalized B cells harvested from the blood of allergic donors, in inducing basophil degranulation. The hIgE mAb used in this study recognize different epitopes on the major HDM allergen Der p 2 and cat (*Felis domesticus*) allergen Fel d 1, as well as peanut (*Arachis hypogaea*) allergen Ara h 2. These clinically relevant allergens were used as model allergens representative of major inhalant and food allergens. Given the prevalence of sensitization to these major allergens: 80-90% of people allergic to HDM are sensitized to Der p 1 or Der p 2; 30.3% of cat allergic patients suffer from asthma attacks upon cat allergen exposure, with Fel d 1 alone constituting 60-90% of allergic activity in cat extracts (10); and Ara h 2 sensitization is present in 40-90% of patients with peanut allergy (11–13). Determining the biological activity of these hIgE mAb is highly relevant to the investigation of allergic disease. For this purpose, rat basophilic leukemia (huRBL) cells transformed with the human high-affinity receptor for IgE (FcϵR1) were utilized, to determine the capacity of the hIgE mAb to sensitize huRBL cells and subsequently to be stimulated with their respective allergens to induce IgE crosslinking and degranulation. We further conducted a specificity screening of the hIgE mAb using homologous allergens with high sequence similarity and compared usage of hIgE mAb to serum sensitization to highlight their additive value in the characterization of biosafety in allergen products.

## 2 Methods

### 2.1 Selection of hIgE mAb

hIgE mAb specific for either Der p 2, Fel d 1 or Ara h 2 were produced according to the previously described protocol (4). The hIgE mAb were derived from several fusions using B cells from HDM, cat or peanut allergic donors who presented to the Vanderbilt University Medical Center allergy clinic and were validated by ImmunoCAP, ELISA and immunoblotting for IgE reactivity. Further details of the donor population and the hIgE mAb panels will be published elsewhere (B. Smith et al, manuscript in preparation). Three anti-Der p 2 hIgE mAb (1B8, 2G1 and 2F10), three anti-Fel d 1 hIgE mAb (6A1, 1B7and 11A12) and five anti-Ara h 2 hIgE mAb (9H11, 13D9, 11F10, 38B7 and 26C3) with distinct epitope specificities were combined in pairs according to their corresponding allergen specificity (e.g., two anti-Der p 2 mAb specific to two distinct epitopes formed one pair) (**Supplementary Table 1**). Pairs were then used to investigate the biological activity of the hIgE mAb.

### 2.2 Passive sensitization of huRBL cells

HuRBL cell mediator release assay was performed as previously described (14). In brief, huRBL cells were passively sensitized overnight with either hIgE mAb or an allergic donor sera pool, and then stimulated with allergen to induce IgE crosslinking and mediator release. The substrate 4-Methylumbelliferyl b-D-glucuronide dihydrate (4MUG, Sigma, Darmstadt, Germany) was used to measure levels of β-hexosaminidase in culture supernatants as surrogate marker for mediator release.

For sensitization, hIgE mAb were combined in a 1:2 ratio and adjusted to the same specific IgE (sIgE) concentration (kU/L) diluted in huRBL medium (MEM with Earl’s salts without L-Glutamine, Sigmal-Aldrich (M8042), supplemented with: 5% FCSi, 4 mM L-Glutamine and G418). Final concentration of hIgE mAb for sensitization ranged between 0.1-250 kU/L of sIgE, was titrated either in 1:3 or 1:2 dilution steps on the cells. HuRBL cells sensitized with the hIgE mAb were incubated overnight at 37°C and 7% CO_2_ to achieve binding to FcϵR1. Details for sensitization using patients’ sera can be found in the supplementary information (**Supplementary Table 2, 3**).

### 2.3 Allergen stimulation of sensitized huRBL cells

For huRBL cell stimulation, HDM allergens natural and recombinant Der p 2 and Der f 2 (InBio, Charlottesville, VA, USA), recombinant *Blomia tropicalis* antigen Blo t 2 (15), *Dermatophagoides pteronyssinus* extract (*Dp* extract, Stallergenes GmbH, Kamp-Lintfort, Germany) and LoTox *Dp* extract (InBio) were compared. Cat allergen stimulations used Fel d 1 (natural and recombinant) (InBio) and a cat hair extract (CHE) (Stallergenes GmbH). For peanut allergens, natural Ara h 2 and Ara h 6, and an *Arachis hypogaea* (*Ah*) extract (InBio) were compared (**Supplementary Table 4**). All allergen/extract concentrations were adjusted to the concentration of the hIgE mAb target allergen within the extract (e.g., the used *Dp* extract concentration contained the same amount of Der p 2 as the purified Der p 2) based on relative quantification using SDS-PAGE (**Supplementary Figure 1, 2**). The concentration of allergen homologs was also adjusted to the hIgE mAb target allergen concentration. Antigen concentrations in the assays ranged between 10^1^ and 10^-8^ µg/mL.

### 2.4 Controls for mediator release assays

As controls, at least two wells of non-stimulated sensitized huRBL cells (sensitized either with sera pool or mAb), two wells for background (unsensitized, unstimulated cells, only treated with medium), and two wells for the maximal lysis control (complete cell lysis using 1% Triton X-100) were included. As additional controls, individual hIgE mAb and hIgE mAb in combination (in equal parts) with hIgE mAb 1E7 specific to an unrelated allergen (*Gallus domesticus* egg white antigen, Gal d 4) were used to sensitize huRBL cells. Cells sensitized with individual hIgE mAb and hIgE mAb paired with 1E7 were stimulated with either rDer p 2, rFel d 1 or nAra h 2 (according to the hIgE mAb allergen specificity) as described above.

### 2.5 Data analysis and statistics

All statistical analyses and visualizations of the data were done using Microsoft Excel (version 2016, Microsoft) and GraphPad Prism (version 9, GraphPad). After background subtraction, results were expressed as percentage of the maximal lysis. To control for assay-to-assay variability due to differences in cell responsiveness and maximal lysis, min-max normalization was performed for each assay. HuRBL assays were performed in technical duplicates and repeated at least twice. Results showing statistical significance were repeated at least a total of three times. Error bars represent the standard deviation of duplicates. Limit of quantification (LOQ) was determined for mediator release curves (mean of baseline values + 10x standard deviation of baseline values) and was used to define significant mediator release. Area-under-the-curve (AUC) analysis (covering all concentration ranges) was performed by transforming the mediator release data to X = log (X), and the baseline for the AUC was set to include the lowest transformed value per data set. AUC of 0% mediator release was defined for each AUC analysis. Significant differences between the various combinations of hIgE mAb as well as between the hIgE mAb and the sera pool were analyzed with ordinary one-way ANOVA with Tukey’s multiple comparisons tests, and unpaired t-tests. Significance was represented with asterisks: *p* < 0.05 (*), *p* < 0.01 (**), *p* < 0.001 (***), and *p* < 0.0001 (****).

## 3 Results

### 3.1 HuRBL cells sensitized with combinations of hIgE mAb are able to induce mediator release upon allergen stimulation

**Figure 1:**
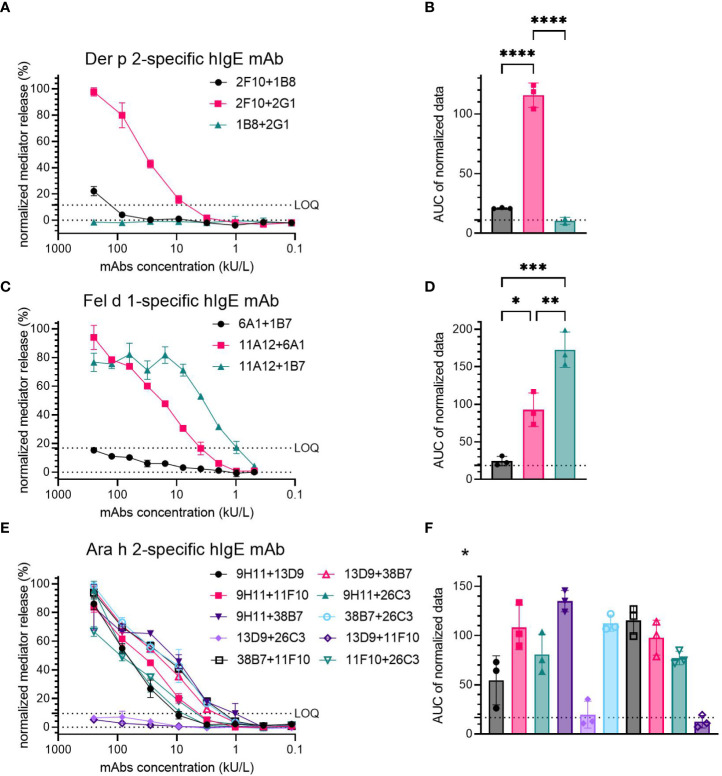
Capacity of hIgE mAb to Der p 2 **(A, B)**, Fel d 1 **(C, D)** and Ara h 2 **(E, F)** for sensitization of huRBL cells to induce degranulation upon allergen stimulation. Sensitized cells were stimulated using 0.5 µg/mL of either rDer p 2, rFel d 1 or nAra h 2. Representative release curves depicting the normalized mediator release are shown **(A, C, E)**. LOQ is shown in all mediator release curve graphs. AUC of normalized mediator release values was performed, and AUC for 0% mediator release is expressed as dotted line **(B, D, F)**. Ordinary one-way ANOVA with Tukey’s multiple comparisons was used to determine differences between combinations; anti-Der p 2 mAb, **(B)**; anti-Fel d 1, **(D)**; and anti-Ara h 2, **(F)**. *Statistical significance for anti-Ara h 2 mAb can be found in **Supplementary Table 5**. p < 0.05 (*), p < 0.01 (**), p < 0.001 (***), and p < 0.0001 (****).

Der p 2-specific hIgE mAb were tested in three pairs (2F10 + 1B8, 2F10 + 2G1 and 1B8 + 2G1), from which sensitization with 2F10 + 2G1 resulted in high mediator release (100% at 250 kU/L hIgE mAb concentration), with release values exceeding the LOQ already at a mAb concentration of 10 kU/L (**Figure 1A**). Combination 2F10 + 1B8 induced low mediator release (~20% at 250 kU/L) just above the LOQ at the highest concentration, whereas the combination 1B8 + 2G1 did not result in mediator release. A significant difference was found when comparing the AUC of combination 2F10 + 2G1 (average AUC=115.63), to that of 2F10 + 1B8 (average AUC=21.04) (*p*<0.0001) and 1B8 + 2G1 (average AUC=10.46) (*p*<0.0001) (**Figure 1B**).

Three pairs of Fel d 1-specific hIgE mAb were also tested (6A1 + 1B7, 11A12 + 6A1 and 11A12 + 1B7). Two combinations, 11A12 + 6A1 and 11A12 + 1B7, produced significant mediator release (>80% at 250 and 62.5 kU/L, respectively); 6A1 + 1B7 induced mediator release below the LOQ (~15% at 250 kU/L) (**Figure 1C**). AUC analysis demonstrated a higher degree of mediator release with the 11A12 + 1B7 combination (average AUC=172.7), than that induced by the 11A12 + 6A1 combination (average AUC=92.7) (**Figure 1D**). Average AUC of mediator release curve produced by combination 6A1 + 1B7 was 24.3, around the AUC of 0% mediator release. A significant difference in the induced mediator release was found when comparing all combinations to each other (6A1 + 1B7 vs. 11A12 + 6A1 (*p*=0.0109); 6A1 + 1B7 vs. 11A12 + 1B7 (*p*=0.0002); 11A12 + 6A1 vs. 11A12 + 1B7 (*p*=0.0051)).

Of the ten possible combinations of Ara h 2-specific hIgE mAb, four combinations (9H11 + 26C3, 38B7 + 26C3, 38B7 + 11F10 and 13D9 + 38B7) induced high mediator release (>90% at 250 kU/L) (**Figure 1E**), four combinations (9H11 + 13D9, 9H11 + 11F10, 9H11 + 38B7 and 11F10 + 26C3) induced moderate mediator release (between 60% and 90% at 250 kU/L) and two combinations (13D9 + 26C3 and 13D9 + 11F10) failed to induce significant mediator release above the LOQ. The combinations inducing the highest mediator release had an AUC >95, and the intermediate and low inducing combinations had an AUC of 50-80 and <20, respectively (**Figure 1F**). Statistically significant differences were observed between several Ara h 2-specific hIgE mAb combinations. A table containing those combinations and their *p*-values can be found in the **Supplementary Material (Table 5)**.

### 3.2 Are combinations of hIgE mAb recognizing at least two distinct epitopes needed to induce mediator release?

**Figure 2:**
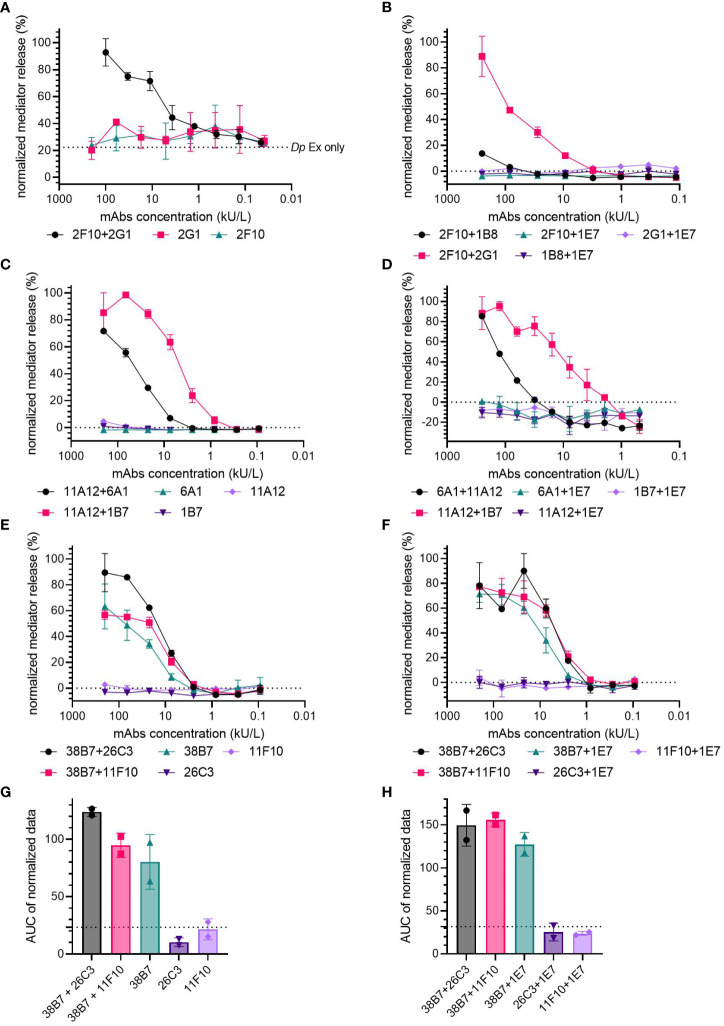
Are two hIgE mAb recognizing the same allergen required to induce mediator release in huRBL cells? Cells were sensitized with a starting concentration of 200 kU/L of of anti-Der p 2 **(A, B)**, anti-Fel d 1 **(C, D)** and anti-Ara h 2 hIgE mAb **(E-H)**, followed by a 1:3 titration and stimulated to induce degranulation with 1 µg/mL of *Dp* extract **(A)**, rDer p 2 **(B)**, rFel d 1 **(C, D)** or nAra h 2 **(E-H)**. Cells were sensitized with an individual hIgE mAb **(A, C, E)** or with an individual hIgE mAb paired with hIgE mAb to Gal d 4 (clone IE7) **(B, D, F)** and compared to a mediator-release-triggering hIgE mAb combination. Dotted line in **(A)** represents *Dp* extract background signal. AUC of the normalized mediator release of the Ara h 2 hIgE mAb controls, and AUC for 0% mediator release is expressed as dotted line **(G, H)**.

To control for non-specific spontaneous degranulation of the huRBL cells by individual mAb sensitization, the hIgE mAb were either screened individually or in combination with hIgE mAb 1E7, specific for an unrelated allergen (Gal d 4), for mediator release upon stimulation with either Der p 2, Fel d 1 or Ara h 2. Mediator release curves from either control were compared to curves from combinations which showed a capacity to induce mediator release (**Figure 2**).

Der p 2- (**Figure 2A, B**) and Fel d 1-specific hIgE mAb (**Figures 2C, D**) did not result in any relevant mediator release either in the individual sensitization or in combination with the Gal d 4-specific hIgE mAb, when compared to pairs of mAb specific to the same allergen. Non-sensitized cells incubated with a high concentration of *Dp* extract (1 µg/mL of corresponding Der p 2 concentration) experienced an average 22.4% spontaneous mediator release (dotted line in **Figure 2A**). Spontaneous mediator release in unsensitized cells occurred when they were stimulated with high concentrations of either *Dp* extract or LoTox *Dp* extract (1-0.01 µg/mL) (**Supplementary Figure 3**).

Ara h 2-specific hIgE mAb 11F10 and 26C3 did not lead to the induction of mediator release in huRBL cells when used for individual hIgE mAb sensitization or in combination with 1E7 (**Figure 2E, F**). Unexpectedly, hIgE mAb 38B7 induced mediator release on its own; 63.4% and 71.4% maximum mediator release, for individual and Gal d 4-specific hIgE mAb-paired sensitizations, respectively. Individually, mAb 38B7 induced comparable mediator release to the high performing combinations 38B7 + 26C3 (89.6%) and 38B7 + 11F10 (56.8%), as well as when paired with the Gal d 4 hIgE mAb (90.0% produced by 38B7 + 26C3 and 77.3% by 38B7 + 11F10), also following a similar release curve. The hIgE mAb 38B7 had a higher average AUC in both the individual (AUC=80.31) and Gal d 4-paired (AUC=127.25) controls, when compared to 26C3 individual (AUC=10.33, *p*=0.0134) and Gal d 4-paired (AUC=25.44, *p*= 0.0038) and 11F10 individual (AUC=21.56, *p*=0.0277) and Gal d 4-paired controls (AUC=23.68, *p*=0.0036), with both having an average AUC around 0% mediator release (dotted line, **Figure 2G, H**). Only a slight difference was found between the AUC of the individual 38B7 (AUC=80.31) and that of the 38B7 + 26C3 (AUC=149.55) and 38B7 + 11F10 combinations (AUC=155.95), and hardly any difference when in combination with 1E7 (AUC=127.25).

### 3.3 The hIgE mAb are highly specific for their target allergen

The specificity of hIgE mAb towards their target allergen was investigated by comparing the ability of other antigens from the same or related sources to induce hIgE crosslinking and mediator release. Combinations producing high mediator release were used to sensitize huRBL cells (2F10 + 2G1 for Der p 2-specific mAb; 11A12 + 6A1 and 11A12 + 1B7 for Fel d 1-specific mAb; and 38B7 + 11F10, 38B7 + 13D9 and 11F10 + 26C3 for Ara h 2-specific mAb).

**Figure 3:**
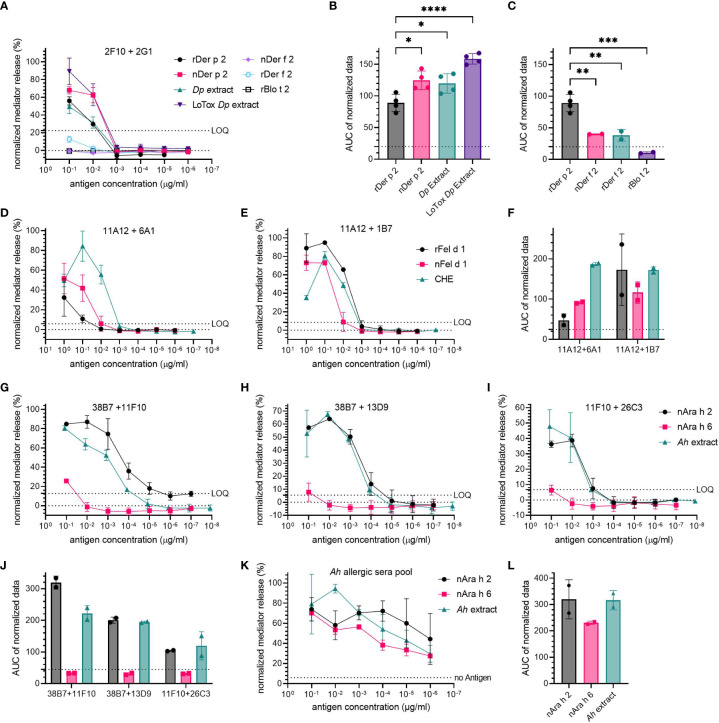
High specificity of hIgE mAb for target allergen. Cells were sensitized with 30 kU/L of hIgE mAb and stimulated to induce degranulation using a starting concentration of 0.1 µg/mL rDer p 2 and 1 µg/mL rFel d 1 and nAra h 2, followed by a 1:10 titration. Concentration for extracts were adjusted to the respective antigen (e.g., Der p 2, Fel d 1 and Ara h 2). Combination 2F10 + 2G1 was used for anti-Der p 2 hIgE mAb **(A-C)**; combinations 11A12 + 6A1 and 11A12 + 1B7 were used for anti-Fel d 1 hIgE mAb **(D-F)**; and 38B7 + 11F10, 11F10 + 26C3 and 38B7 + 13D9 were used for anti-Ara h 2 hIgE mAb **(G-J)**. LOQ is provided as dotted line. A peanut allergic sera pool was also tested for its specificity **(K)**, average sIgE for Ara h 2 and for Ara h 6 were determined to be 12.75-13.14 kU/L. AUC of mediator release curves is shown, with AUC for 0% mediator release as dotted line **(B, C, F, J, L)**. An ordinary one-way ANOVA with Tukey’s multiple comparisons was used to determine statistical significance between different HDM antigens **(B, C)**.

Der p 2-specific mAb were stimulated with either natural or recombinant Der p 2, as well as with two distinct HDM extracts (*Dp* extract and LoTox *Dp* extract). The Der p 2 homologs Der f 2 (natural and recombinant) and rBlo t 2 were used for the specificity assessment of the Der p 2-specific hIgE mAb (**Figures 3A-C**). A high specificity of Der p 2-specific hIgE mAb to Der p 2-containing sources was observed (mediator release at 0.1 µg/mL of corresponding Der p 2 concentration: 56% for rDer p 2, 67.9% for nDer p 2, 49.4% for *Dp* extract and 89.3% for LoTox *Dp* extract). The Der p 2-homolog allergens induced hardly any relevant mediator release (12.7% by rDer f 2, -0.58% by nDer f 2 and 0.33% by rBlo t 2, each allergen at 0.1 µg/mL, all below the LOQ). Compared to rDer p 2 (88.9 AUC), all natural allergen preparations had a significantly higher AUC (nDer p 2 AUC=124.6, *p*=0.0119; *Dp* extract AUC=119.7, *p*=0.0293; and LoTox *Dp* extract AUC=158.5, *p*<0.0001). Stimulation with rDer p 2 resulted in a 2.2-15.1-fold significantly higher AUC compared to the Der p 2 homologs (rDer f 2 AUC=38.2, *p*=0.0062; nDer f 2 AUC=40.0, *p*=0.0052; and rBlo t 2 AUC=10.5, *p*=0.0005).

The cells sensitized with Fel d 1-specific hIgE mAb were stimulated with either recombinant or natural Fel d 1, or a commercial cat hair extract (CHE) (**Figures 3D-F**). The combination 11A12 + 6A1 produced the lowest mediator release with a maximum release of 32.4% for rFel d 1 (AUC=47.1) and 51.7% for nFel d 1 (AUC=91.4) (both at 1 µg/mL), and the highest mediator release of 84.4% for 0.1 µg/mL of CHE (AUC=186.7). For 11A12 + 1B7, all three Fel d 1-containing samples resulted in relatively similar mediator release dose-response curves, with a maximum release of 95% and 80.6% at the corresponding Fel d 1-concentration of 0.1 µg/mL for rFel d 1 (AUC=172.9) and CHE (AUC=171.9), respectively, and 73.1% for 1 µg/mL of nFel d 1 (AUC=116.2).

Regarding the specificity of the Ara h 2-specific hIgE mAb, nAra h 2, nAra h 6 and an *Ah* extract were used for basophil stimulation (**Figures 3G–J**). Sensitization with the two Ara h 2-specific hIgE mAb combinations 38B7 + 11F10 and 38B7 + 13D9 resulted in a high maximum mediator release (> 60%) when stimulated with nAra h 2 or *Ah* extract at 0.1 µg/mL of corresponding Ara h 2 concentration, whereas sensitization with 11F10 + 26C3 resulted in a slightly lower maximum (38.6% with nAra h 2 and 47.8% with *Ah* extract at 0.1 µg/mL). Stimulation with nAra h 6 induced a low mediator release, only exceeding the LOQ for 38B7 + 11F10 at the highest concentration with 25.7%. The AUC of the mediator release induced by nAra h 2 was on average only 1.1-fold higher than that of the *Ah* extract. When compared to nAra h 2, the AUC of nAra h 6 stimulated cells was 9.7-, 6.6- and 3.3-fold lower in combinations 38B7 + 11F10, 38B7 + 13D9 and 38B7 + 11F10, respectively. AUC of nAra h 6 was lower than the *Ah* extract by 6.7-, 6.4- and 3.8-fold in 38B7 + 11F10, 38B7 + 13D9 and 11F10 + 26C3, respectively. When sensitizing huRBL cells with an allergic donor sera pool, it was not possible to discriminate between the mediator release induced by nAra h 2 and nAra h 6 or *Ah* extract (**Figures 3K, L**).

### 3.4 Sensitization with hIgE mAb is comparable to sensitization using allergic donor sera pools

**Figure 4:**
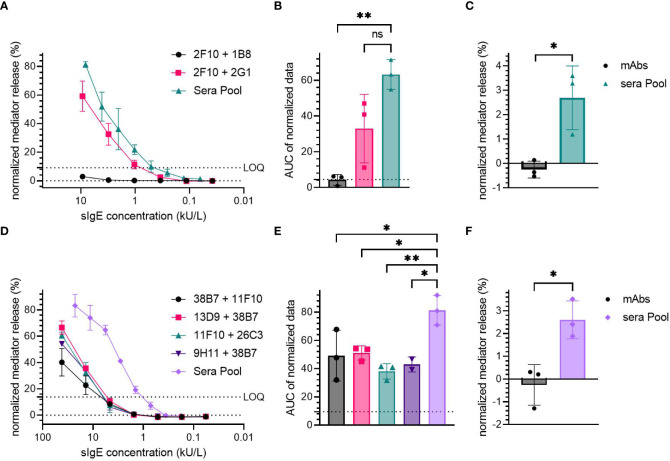
Comparison of hIgE mAb sensitization to human sera pool sensitization. Cells were sensitized with either hIgE mAb combination or a sera pool; Der p 2-specific hIgE mAb: **(A-C)**, Ara h 2-specific hIgE mAb: **(D-F)**. HIgE mAb had a starting concentration of 9.3 kU/L for Der p 2 and 41.67 kU/L for Ara h 2 specific mAb, followed by a 1:3 titration. HDM allergic donor sera pool had a starting sIgE concentration of 9.3 kU/L, and the peanut allergic donor sera pool had a starting sIgE concentration of 22.8 kU/L. Cells were stimulated with 0.1 µg/mL of either rDer p 2 or nAra h 2 in order to induce degranulation. LOQ is shown in representative mediator release curves. An ordinary one-way ANOVA with Tukey’s multiple comparisons was used on AUC data of the normalized mediator release values to determine significant differences **(B, E)**. Dotted line was used to express AUC for 0% mediator release. Spontaneous degranulation for each sensitization condition without antigen stimulation were compared, and their statistical differences were determined using an unpaired t-test **(C, F)**. p < 0.05 (*), p < 0.01 (**). ns, not significant.

The sensitization of huRBL cells using either the Der p 2-specific (2F10 + 2G1 and 2F10 + 1B8, **Figures 4A-C**) or Ara h 2-specific hIgE mAb (38B7 + 11F10, 38B7 + 13D9, 11F10 + 26C3 and 9H11 + 38B7, **Figures 4D-F**) was compared to the sensitization with an allergic donor sera pool.

The Der p 2-specific hIgE mAb combination 2F10 + 2G1 produced comparable mediator release at similar sIgE concentrations (~60% maximum mediator release at 9.26 kU/L), to that produced by the sera pool from HDM allergic donors (>80% at a sIgE concentration of 8.15 kU/L). No significant difference was observed between the AUC of the high-release combination 2F10 + 2G1 (AUC=33) and the sera pool (AUC=63.24).

Sensitization using the sera pool derived from peanut allergic patients induced a high mediator release, > 80% at the average sIgE concentration of 22.78 kU/L, while Ara h 2-specific hIgE mAb combinations produced > 40% mediator release at a concentration of 41.67 kU/L (**Figure 4D**). The average AUC of all Ara h 2 hIgE mAb combinations (49.16 for 38B7 + 11F10, 51.07 for 13D9 + 38B7, 38.11 for 11F10 + 26C3 and 42.95 for 9H11 + 38B7) was significantly lower than of the sera pool (AUC=81.25); with *p*-values of 0.0296, 0.0408, 0.0050 and 0.0206 for 38B7 + 11F10, 13D9 + 38B7, 11F10 + 26C3, and 9H11 + 38B7 compared to the sera pool, respectively.

Both the sera pools from HDM and peanut allergic subjects were found to induce significantly higher spontaneous degranulation in the non-allergen-stimulated control cells when compared to the cells sensitized with the hIgE mAb combinations (approximately 2.6% for both HDM and peanut condition, vs. ~0% for hIgE mAb, **Figures 4C, F**). The sera pools tended to be cytotoxic at higher sIgE concentrations (>1 kU/L, **Supplementary Figure 4**), whereas sensitization with hIgE mAb did not result in cytotoxic effects.

## 4 Discussion

Here, we demonstrate the biological activity of hIgE mAb, based on their capability to induce basophil degranulation upon allergen stimulation. The location and proximity of hIgE mAb epitopes on the allergen determines the capacity of the allergen to cross-link IgE, and therefore to induce mediator release. Not all the IgE mAb pairs are expected to activate the basophils. The mediator release results reported here are in agreement with the location of the areas containing IgE antibody binding epitopes, which were identified by NMR for 2F10, 2G1, 1B8 and 5D10 hIgE mAb (4), and identified in detail by X-ray crystallography for the IgE mAb 2F10, the first reported structure of an IgE mAb in complex with an allergen (Der p 2) (5). As expected, mediator release was not induced in basophils sensitized with hIgE mAb that recognize overlapping epitopes (1B8 and 2G1), whereas it was strongly induced in basophils sensitized with combinations of non-overlapping IgE mAb (2F10 and 2G1). These results support the observations from a mouse model of passive systemic anaphylaxis, showing that both Der p 2-specific hIgE mAb combinations, 2F10 + 2G1 and 2F10 + 1B8, triggered anaphylaxis when compared to the negative controls, although with different potency, also observed for mediator release: the combination 2F10 + 1B8 exceeded the LOQ only at the highest mAb concentration. Based on these results, the combinations 6A1 + 1B7 (Fel d 1-specific hIgE mAb) and 13D9 + 26C3 and 13D9 + 11F10 (Ara h 2-specific hIgE mAb) are composed of mAb that might bind to overlapping epitopes.

Although 38B7 has previously been shown to induce degranulation in combination with another anti-Ara h 2 hIgE mAb (16), we observed here that 38B7 induces crosslinking without the need of another mAb with a different epitope specificity. One can speculate that mAb 38B7 binds the repeated motif DPSYP^OH^S on Ara h 2, a peptide reported to have a high allergenicity that suggests that it could be simultaneously bound by at least two IgE antibodies (17). Bernard et al., found that mast cell degranulation can be induced by any peptide with more than one DPSYP^OH^S motif, even with a molecular weight as low as 3kDa. This DPSYP^OH^S motif is not found in Ara h 6, which has a high degree of sequence similarity (~55%) with Ara h 2 (**Supplementary Figure 5**) (17). Since the remaining sequence of Ara h 2 is rather conserved among Ara h 6, we hypothesize that the epitope specificity of 38B7 is decisive for the exclusive basophil activation by Ara h 2. Thus, the specificity of 38B7, currently being investigated by X-ray crystallography, will help to provide more structural information on this immunodominant IgE epitope on Ara h 2.

Dimerization was described for certain allergens leading to IgE crosslinking by providing two IgE epitopes (18–20). Dimerization of Ara h 2, which could explain the activity seen by the hIgE mAb 38B7, has not been reported. None of the Der p 2-specific or Fel d 1-specific hIgE mAb showed the capacity to induce mediator release on their own, although Der p 2 was reported to partially dimerize naturally at high concentrations (5). SDS-PAGE of rDer p 2 or nDer p 2 used in this study did not show dimers (**Supplementary Figure S1**).

Regarding the specificity screening, the sensitization of huRBL cells with the hIgE mAb proved to be highly specific when comparing stimulations with the respective allergens and homologs thereof. Neither Der f 2 nor Blo t 2, having a sequence similarity of 88% and 40.6%, respectively (4, 5), induced relevant degranulation in cells sensitized with 2F10 and 2G1. Comparing the epitope preferences of these two antibodies highlights that only three amino acids in the Der f 2 sequences are altered in comparison to Der p 2 (**Supplementary Figure S6**). Furthermore, 2F10 was previously shown to bind Der f 2 to the same extent in ELISA (5). By using serum for sensitizing the cells, it was not possible to distinguish the biological activity of homologs. Co-sensitization to various major allergens and polyclonality are two factors why sera pools are neither allergen- nor epitope-specific (21). The high specificity for discriminating their target allergen from homologs is a great advantage of the hIgE mAb, which is of utmost importance in characterizing allergen extracts for allergen immunotherapy to guarantee a standardized biological activity in different extract batches; especially for food allergens such as Ara h 2 and Ara h 6 that induce anaphylactic reactions (21, 22).

In the case of the Der p 2-specific hIgE mAb, 2F10 + 2G1 induced comparable mediator release as the HDM allergic sera pool. On the other hand, although eight combinations of Ara h 2 specific hIgE mAb were found to produce significant mediator release, none of them were able to induce comparable mediator release as the peanut allergic sera pool. The polyclonal character of IgE in human serum is likely the reason for the higher release as higher clonality results in increased mediator release (8). Future comparisons between hIgE mAb and allergic sera pool should be done combining all five mAb specific to Ara h 2, to see if there is an additive effect by providing additional paratopes.

The significantly higher average spontaneous mediator release observed in the allergic sera pools is worth noting. This is not an unexpected finding, since it has been reported that human serum sensitization has cytotoxic effects and can induce a spontaneous non-antigen-stimulated degranulation in huRBL cells (6, 23, 24). Unlike hIgE mAb, human serum standardization in assays to determine the biological activity of allergens is more complex due to its limited quantities and availability, reproducibility, polyclonality/allergen-specificity, and potential cytotoxicity resulting in allergen-independent spontaneous degranulation.

For these reasons, hIgE mAb could serve as a suitable alternative to the currently used human reference serum in quality control and diagnostic assays (25). Additionally, hIgE mAb could be useful in developing a more patient-friendly allergy immunotherapy by designing hypoallergenic vaccines by specifically altering the symptom-associated IgE epitopes while keeping the treatment-relevant IgG_1_ and IgG_4_ epitopes of the target allergen intact.

## Data availability statement

The original contributions presented in the study are included in the article/**Supplementary Material**. Further inquiries can be directed to the corresponding author.

## Ethics statement

The studies involving human participants were reviewed and approved by Institutional Review Board of the University of Colorado, Denver and institutional review board (CREATE). Written informed consent to participate in this study was provided by the participants’ legal guardian/next of kin.

## Author contributions

The study was conceived and designed by MC, AP, RR and LA. GP-C devised and performed experiments, wrote the manuscript and created figures. BS, AP, SS and MC validated the hIgE mAb for use in the study and provided purified allergens. MS, HW and SV conducted experiments. RvR provided sera from allergic patients. LA devised, performed and supervised experiments, and led the study. All authors read, edited and approved the manuscript.
