# Corrigendum: Biological activity of human IgE monoclonal antibodies targeting Der p 2, Fel d 1, Ara h 2 in basophil mediator release assays

**DOI:** 10.3389/fimmu.2023.1296381

**Published:** 2023-10-10

**Authors:** Glorismer Pena-Castellanos, Bryan R. E. Smith, Anna Pomés, Scott A. Smith, Maria A. Stigler, Hannah L. Widauer, Serge A. Versteeg, Ronald van Ree, Martin D. Chapman, Lorenz Aglas

**Affiliations:** ^1^ Department of Biosciences and Medical Biology, University of Salzburg, Salzburg, Austria; ^2^ InBio, Charlottesville, VA, United States; ^3^ Department of Medicine, Vanderbilt University Medical Center, Nashville, TN, United States; ^4^ Department of Experimental Immunology, Amsterdam University Medical Centers, Amsterdam, Netherlands; ^5^ Department of Otorhinolaryngology, Amsterdam University Medical Centers, Amsterdam, Netherlands

**Keywords:** human IgE monoclonal antibodies, mediator release assay, Der p 2, Fel d 1, Ara h 2, allergy diagnosis

In the published article, there was an error in Supplementary Table 1. In the originally submitted Supplementary Table 1, the names of the hIgE mAbs specific for the Der p 2 allergen were mixed up. Additionally, the concentrations reported for the total IgE concentrations for the Fel d 1 specific hIgE mAbs in this table were incorrect (these were the concentrations of sIgE found for the Cat dander extract not the total IgE concentration). Total IgE concentrations for any of the hIgE mAbs were not used for any of the experiments and were only included for the sake of completion.

The correct Supplementary Table 1 has been updated in the original article.

The authors apologize for this error and state that this does not change the scientific conclusions of the article in any way.

