# Supplementary material for: Biological activity of human IgE monoclonal antibodies targeting Der p 2, Fel d 1, Ara h 2 in basophil mediator release assays
Source: Front Immunol. 2023 May 9;14:1155613. doi: 10.3389/fimmu.2023.1155613 (PMC10203493; doi:10.3389/fimmu.2023.1155613)
Supplement: Supplementary file 1 [file DataSheet_1.docx]

Supplementary Material

Biological activity of human IgE monoclonal antibodies targeting Der p 2, Fel d 1, Ara h 2 in basophil mediator release assays

Glorismer Pena-Castellanos^1^; Bryan R.E. Smith^2^; Anna Pomés^2^; Scott A. Smith^3^; Maria A. Stigler^1^; Hannah L. Widauer^1^; Serge A. Versteeg^4^; Ronald van Ree^4^; Martin D. Chapman^2^; Lorenz Aglas^1^*

^1^University of Salzburg, Department of Biosciences and Medical Biology, Salzburg, Austria;

^2^InBio, Charlottesville, VA, USA;

^3^Vanderbilt University Medical Center, Nashville, TN, USA;

^4^Departments of Experimental Immunology and of Otorhinolaryngology, Amsterdam University Medical Centers, Amsterdam, The Netherlands

*** Correspondence:**
Dr. Lorenz Aglas
ORCID ID: 0000-0002-1236-5934
Department of Biosciences and Medical Biology, University of Salzburg
Hellbrunner Str. 34, A-5020 Salzburg, Austria
Tel.: +43-662-8044-5819
E-mail: [lorenz.aglas@plus.ac.at](about:blank)

# Supplementary Figures and Tables

Supplementary Table 1. List of hIgE mAb specific for Der p 2, Fel d 1, Ara h 2 and Gal d 4.

| **Allergen specificity** | **hIgE mAb** | **sIgE (kU/L)** | **Total IgE (kU/L)** |
| --- | --- | --- | --- |
| Der p 2 | 2G1 | 37,575 | 71,967 |
|  | 2F10 | 19,925 | 46,483 |
|  | 1B8 | 56,300 | 40,500 |
| Fel d 1 | 6A1 | 14,933 | 63,800 |
|  | 1B7 | 18,033 | 67,117 |
|  | 11A12 | 52,667 | 59,967 |
| Ara h 2 | 9H11 | 24,300 | 49,750 |
|  | 13D9 | 69,000 | 50,550 |
|  | 11F10 | 49,250 | 49,200 |
|  | 38B7 | 31,950 | 48,350 |
|  | 26C3 | 37,200 | 51,350 |
| Gal d 4 | 1E7 |  | 50,000 |

Supplementary Table 2. List of peanut allergic donor sera that were combined into a sera pool and their sIgE levels (kU/L) used to sensitized huRBL cell for comparison with hIgE mAb sensitization.

| **Peanut allergic patient** | **sIgE (kU/L)** |
| --- | --- |
| 1 | 52.3 |
| 2 | 544 |
| 3 | 94 |
| 4 | 79.7 |
| 5 | 369 |
| **Average sIgE of pooled sera** | **227.8** |

Supplementary Table 3. List of HDM allergic donor sera that were combined into a sera pool and their sIgE levels (kU/L) used to sensitized huRBL cell for comparison with hIgE mAb sensitization.

| **HDM allergic patient** | **sIgE (kU/L)** |
| --- | --- |
| 1 | 20.4 |
| 2 | 52.9 |
| 3 | 30.2 |
| 4 | 33.6 |
| 5 | 26.9 |
| 6 | 31.3 |
| **Average sIgE of pooled sera** | **32.55** |

Supplementary Table 4. List of antigens used to stimulate IgE crosslinking and their company / product code.

| Species | Antigen (and type) | Company / Product code |
| --- | --- | --- |
| *Dermatophagoides pteronyssinus* | rDer p 2 | InBio / RP-DP2C-1 |
|  | nDer p 2 | InBio / NA-DP2-1 |
|  | *Dp* extract | Stallergenes GmbH/ GB70A01 |
|  | LoTox *Dp* extract | InBio / LTN-DPE-1 |
| *Dermatophagoides farinae* | rDer f 2 | InBio / RP-DF2-1 |
|  | nDer f 2 | InBio / NA-DF2-1 |
| *Blomia tropicalis* | rBlo t 2 | In-house |
| *Felis domesticus** | rFel d 1 | InBio / RP-FD1D-1 |
|  | nFel d 1 | InBio / NA-FD1-1 |
|  | Cat hair extract (CHE) | Stallergenes GmbH/ 375829 |
| *Arachis hypogaea* | nAra h 2 | InBio / LTN-AH2-1 |
|  | nAra h 6 | InBio / LTN-AH6-1 |
|  | *Ah* extract | InBio / LTN-AHRE-4 |


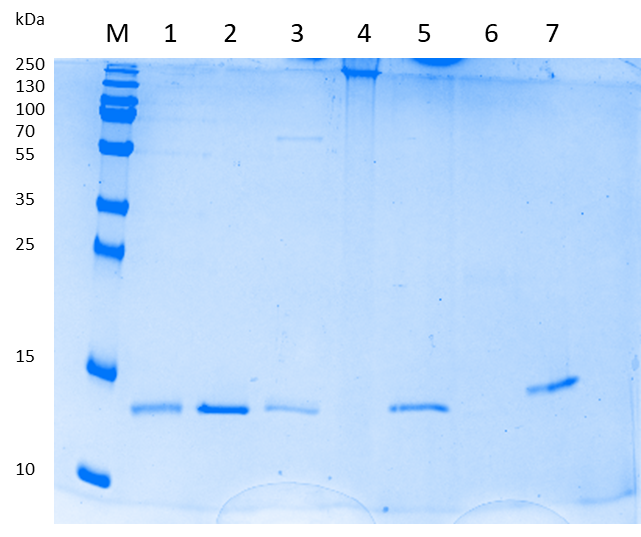


Supplementary Figure 1. SDS page of dust mite antigens. 1 µg of protein per slot was added of each antigen: 1 = nDer p 2; 2 = rDer p 2; 3 = nDer f 2; 4 = *Dp* extract; 5 = rDer f 2; 6 = LoTox *Dp* extract; 7 = rBlo t 2; M = protein marker (PageRuler™ Plus Prestained Protein Ladder, Thermo Scientific, Cat# 26619). Gel was stained with Coomassie Brilliant Blue.


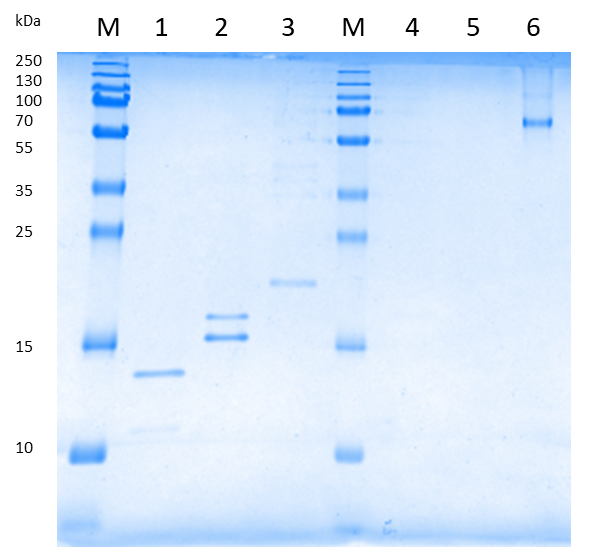


Supplementary Figure 2. SDS page of *Arachis hypogaea* antigens. 2 µg of protein per slot were added of each antigen: 1 = nAra h 6; 2 = nAra h 2; 3 = *Ah* extract; M = protein marker (3µL in slot) (PageRuler™ Plus Prestained Protein Ladder, ThermoScientific, Cat# 26619). Coomassie Brilliant Blue staining was used.

*Due to glycosylation of natural Fel d 1 products (Cat hair extract and natural Fel d 1), the allergen migrates as a diffused band on SDS-page gels, making relative quantification of protein concentration on SDS-page difficult. Therefore, the concentration of products were only determined by allergen specific ELISA (InBio) or WesternBlot (Stallergenes GmbH) using a standard.

Supplementary Table 5. Significant differences in mediator release between pairs of sensitizing hIgE mAb in all Ara h 2-specific hIgE mAb combinations (Fig 1f). ^#^

# Differences were calculated using ordinary one-way ANOVA with Tuckey’s multiple comparisons.

**Passive sensitization of huRBL cells using allergic sera pool**

For sensitization with patients´ sera, specific serum pools were created by combining either six sera from HDM allergic (from CREATE project (1,2) or five sera from peanut allergic donors (kindly provided by Prof. Dr. Stephen Dreskin, Colorado, USA). Ethical approval for using human sera from HDM allergic donors was obtained from the institutional review board of all participating centers and each patient provided written consent. Collection of sera from peanut allergic donors was approved by the Institutional Review Board of the University of Colorado, Denver, all subjects or their guardians signed informed consent and, for minors, assent. Sera from allergic donors were combined in equal volumes from each donor (e.g., 200 µL from each). The quantity of sIgE for Der p 2, Fel d 1 and Ara h 2 in individual sera was determined using ImmunoCAP (Thermo Fisher Scientific, Waltham, MA, USA). The sIgE of the serum pool was determined according to the average of the donor´s individual sIgE levels (Supplementary Table 2-3). To inactivate the complement system within human sera, the sera pools were preincubated with Ag-8 mouse myeloma cells. HuRBL cells were incubated overnight with the complement-inactivated sera pools at 37°C and 7% CO_2_. Potential cytotoxicity induced by either human serum or hIgE mAb was investigated using a cell viability assay with MTT (3-(4,5-Dimethylthiazol-2-yl)-2,5-Diphenyltetrazolium Bromide, Sigma).

Supplementary Figure 3. Comparing background signal induced by *Dp* extracts to mediator release observed in sensitized cells. HuRBL cells were incubated overnight with 30 kU/L of the 2F10+2G1 hIgE mAb combination. For antigen stimulation, a starting concentration of 1 µg/mL of antigen, followed by a 1:10 titration was used. As control, the *Dp* extracts were incubated with the substrate 4MUG, termed “no cells”. These findings indicate that the *Dp* extracts induce a background signal at the highest concentrations.

**Supplementary Figure 4.** Cell viability of huRBL cells sensitized with a cat allergic donor sera pool. Starting average concentration was 10.15 kU/L followed by a 1:2 concentration titration (**a**), cells were not presented with antigen. Less toxic sIgE concentrations (1-0.1 kU/L) resulted in mediator release huRBL cells sensitized with cat allergic donor sera pool upon stimulation with 1 µg/mL rFel d 1 (**b)**. The toxic, high sIgE concentrations did not induce degranulation.


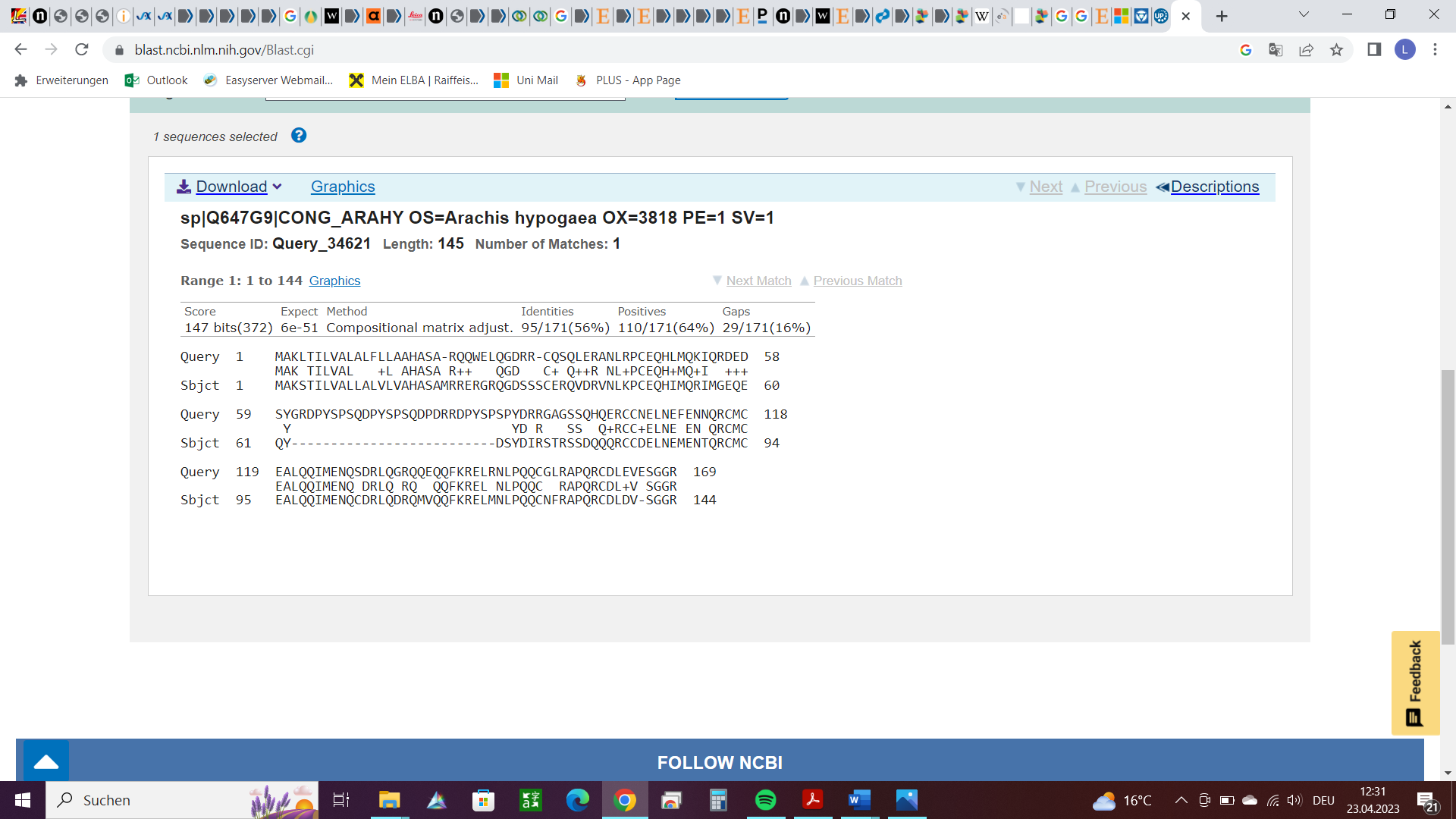


**Supplementary Figure 5.** Amino acid sequence similarity between Ara h 2.0201 (UniProt accession number: Q6PSU2-1), upper line, and Ara h 6.0101 (UniProt accession number: Q647G9), lower line. The repetitive motif DPSYP^OH^S occurring three-times on Ara h 2.0201 but inexistent on Ara h 6.0101 is highlighted with a box.

**Der p 2**

**Der f 2 Blo t 2**


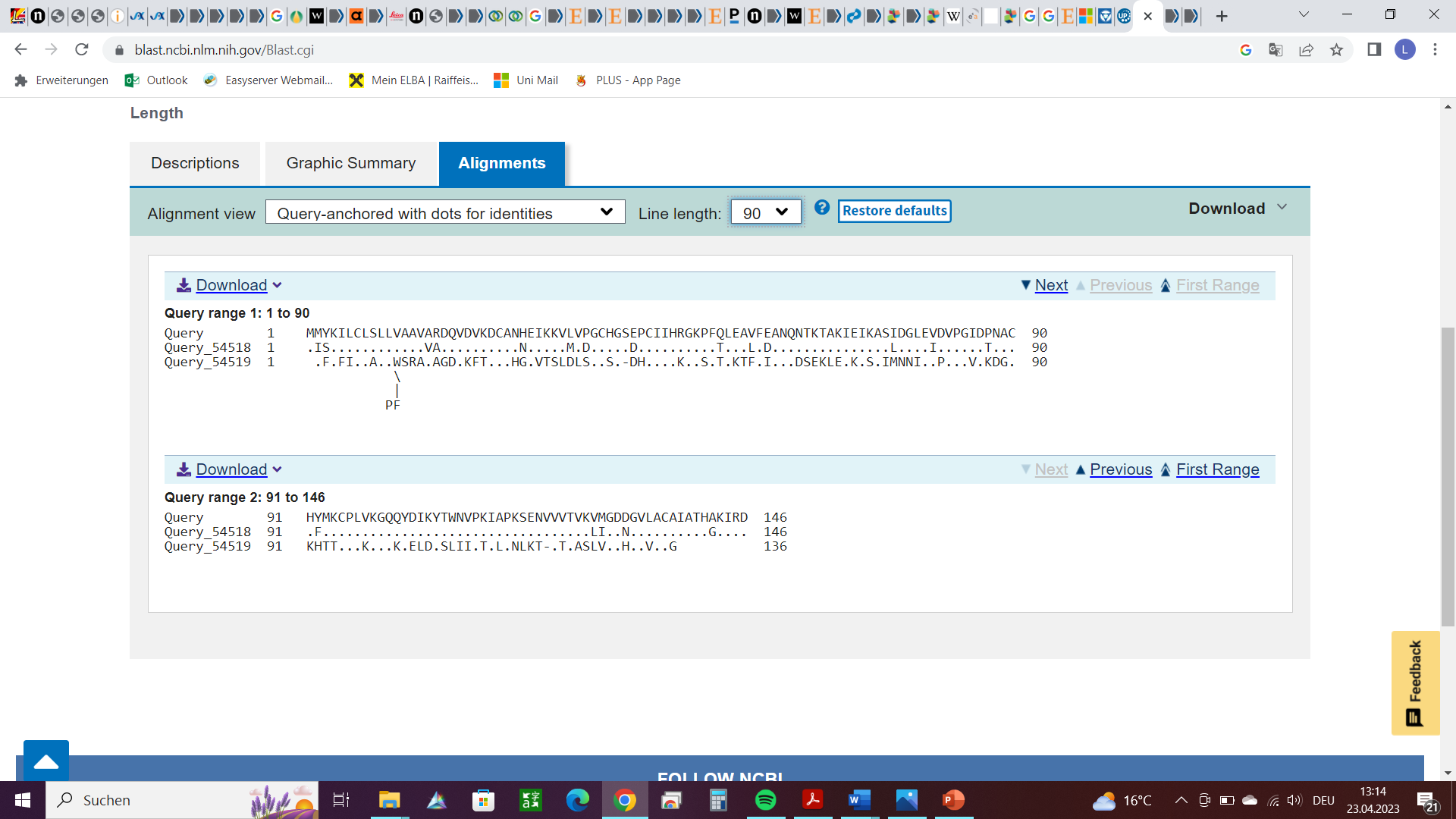

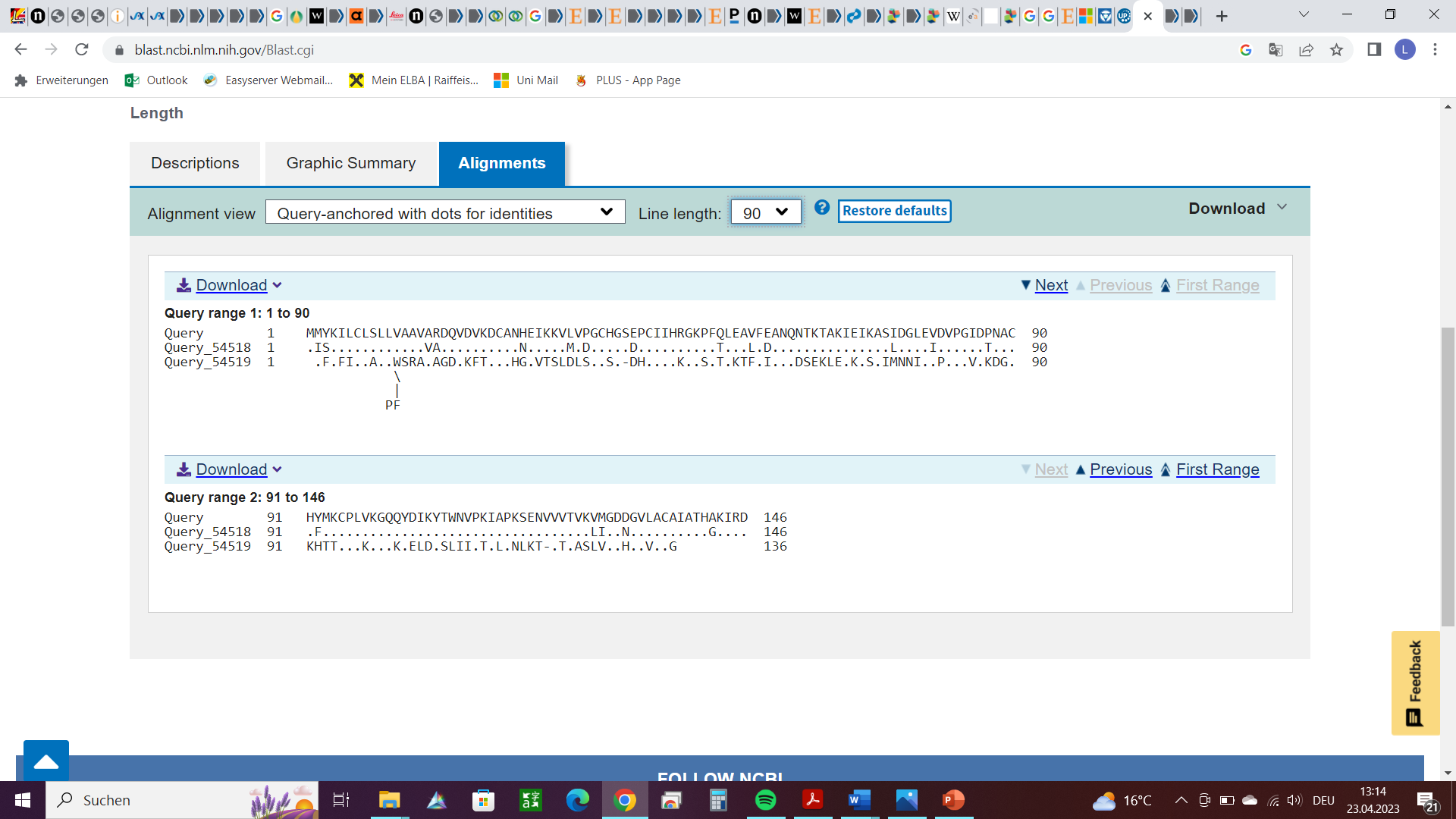


**Der p 2**

**Der f 2 Blo t 2**

**Supplementary Figure 6.** Amino acid sequence similarity between Der p 2.0101 (UniProt accession number: P49278), Der f 2.0101 (UniProt accession number: Q00855), Blo t 2.0101 (UniProt accession number: Q1M2P1). The binding sites for 2F10 and 2G1 on Der p 2.0101 (highlighted with a yellow and green box, respectively) is highly conserved among different species of Dermatophagoides but not in *Blomia tropicalis*, as determined by X-ray crystallography and NMR. Amino acids identical with Der p 2.0101 are highlighted with a (3,4).

**References**

1. van Ree R, Chapman MD, Ferreira F, Vieths S, Bryan D, Cromwell O, et al. The CREATE Project: Development of certified reference materials for allergenic products and validation of methods for their quantification. Vol. 63, Allergy: European Journal of Allergy and Clinical Immunology. 2008. p. 310–26.

2. Chapman MD, Ferreira F, Villalba M, Cromwell O, Bryan D, Becker WM, et al. The European Union CREATE Project: A model for international standardization of allergy diagnostics and vaccines. Journal of Allergy and Clinical Immunology. 2008;122(5).

3. Mueller GA, Glesner J, Daniel JL, Zhang J, Hyduke N, Richardson CM, et al. Mapping Human Monoclonal IgE Epitopes on the Major Dust Mite Allergen Der p 2. The Journal of Immunology. 2020 Oct 15;205(8):1999–2007.

4. Khatri K, Richardson CM, Glesner J, Kapingidza AB, Mueller GA, Zhang J, et al. Human IgE monoclonal antibody recognition of mite allergen Der p 2 defines structural basis of an epitope for IgE cross-linking and anaphylaxis in vivo. PNAS Nexus. 2022;(June):1–12.
